# Interaction of Straw Mulching and Nitrogen Fertilization on Ammonia Volatilization from Oilseed Rape–Maize Rotation System in Sloping Farmland in Southwestern China

**DOI:** 10.3390/plants14010014

**Published:** 2024-12-24

**Authors:** Li Yao, Hong Wang, Haitao Liu, Xie Wang, Yueying Wu, Lin Wang, Honglin Chen, Chaowen Lin

**Affiliations:** Agricultural Resources and Environment Institute, Sichuan Academy of Agricultural Sciences, Chengdu 610066, China; yaolisfri@163.com (L.Y.);

**Keywords:** ammonia volatilization, straw, urease inhibitor, controlled-release urea, organic fertilizer, southwestern China

## Abstract

Ammonia (NH_3_) volatilization caused by urea application has negative implications for human health, environmental quality, and the value of nitrogen fertilizers. It remains to be investigated how management strategies should be adopted to not only reduce NH_3_ volatilization but also improve nitrogen use efficiency (NUE) in the agriculture industry at present. Hence, a two-year field trial, including subplots, was conducted to simultaneously evaluate the effects of mulching treatments (NM: non-mulching; SM: straw mulching) and different fertilizer treatments (U: urea; U + NBPT: urea plus 1% N-(n-butyl) thiophosphoric triamide; U + CRU: the mixture of urea and controlled-release urea at a 3:7 ratio; U + OF: urea plus commercial organic fertilizer at a 3:7 ratio) on NH_3_ volatilization, crop production, and NUE in an oilseed rape–maize rotation system in the sloping farmland of purple soil in southwestern China between 2021 and 2023. Compared with NM + U, NH_3_ volatilization losses under the NM + U + NBPT, NM + U + CRU, and NM + U + OF treatments decreased, on average, by 64.13%, 17.39%, and 15.09% during the oilseed rape growing season but by 64.01%, 11.67%, and 10.13% during the maize growing season, respectively. An average increase in NH_3_ volatilization of 35.65% for the straw-mulching treatment was recorded during the oilseed rape season, while during the maize season, this parameter showed an increase of 10.69%, in comparison to NM + U. With the combination of urea with NBPT, CRU, and organic fertilizer, contrastingly, a reduction in NH_3_ volatilization was achieved under the SM + U + NBPT, SM + U + CRU, and SM + U + OF treatments. When compared with NM + U, the difference in the NUE between the NM + U + NBPT, NM + U + CRU, and NM + U + OF treatments was not significant in the oilseed rape season. The NUE was around 4.27% higher under NM + U + NBPT during the maize season (*p* < 0.05). Compared with NM + U, under the NM + U + NBPT, NM + U + CRU, and NM + U + OF treatments, consistently lower values of yield-scaled NH_3_ volatilization were noted: 13.15–65.66% in the oilseed rape season and 10.34–67.27% in the maize season. Furthermore, SM + U, SM + U + NBPT, SM + U + CRU, and SM + U + OF showed average annual emission factors (AEFs) of 14.01%, 5.81%, 12.14%, and 11.64%, respectively. Overall, straw mulching, along with the application of the mixture of NBPT and urea, was found to be the optimal strategy to effectively reduce the NH_3_ emissions in the purple soil areas of southern China.

## 1. Introduction

Nitrogen (N) is an essential nutrient for crop development in various agricultural ecosystems. N fertilizer application can rapidly boost crop yield in addition to improving food safety. Great N losses and low fertilizer use efficiency, however, may result from the excessive application of chemical N fertilizers. Nitrogen can be lost from the soil through ammonia (NH_3_) volatilization, as well as nitrogen (N_2_), nitrous oxide (N_2_O), and nitrogen dioxide (NO_2_) emissions from leaching and runoff during heavy rainfall events [1,2]. N volatilization loss may occur after applying urea to the soil surface without it being incorporated into the soil, while losses via NH_3_ volatilization may account for 40–60% of the total N fertilizer applied [3,4]. Apart from increasing farm production costs, these high N losses through NH_3_ volatilization have negative ecological impacts such as surface water eutrophication, soil acidification, and acidic precipitation, forming particulate matter like PM 2.5 (particles ≤ 2.5 um in diameter) [4,5]. With the aim of reducing global greenhouse gas emissions, the government of China has set the dual goals of peaking carbon dioxide emissions before 2030 and achieving carbon neutrality by 2060 [6]. Therefore, developing effective management strategies to reduce N losses from farmlands alongside improving N use efficiency (NUE) is essential.

Various soil factors, including soil moisture, texture, pH, and temperature, agricultural measures (the source, type, and rate of fertilizers and tillage practices), and weather variables (precipitation, air temperature, wind, and sunlight), influence ammonia volatilization [7,8,9,10]. The mitigation measures for NH_3_ volatilization from N fertilizers in agricultural systems have been thoroughly studied. The inorganic fertilizers used in China, including urea and the urea-based compound fertilizer, stand out as the major forms of N fertilizers applied to agricultural crops, usually by either surface application or incorporation into the soil [11]. The NH_3_ volatilization resulting from the incorporation of urea into the soil at a depth of 7.5 cm is minimal [12]. However, since it is both time- and labor-saving, the surface application of urea may be the dominant practice under different circumstances, particularly on large farms [11].

With the adoption and improvement of the technologies for fertilizer use reduction and efficiency enhancement in China, Chinese scientists and businesses have devised various successful irrigation, fertilization, and tillage management strategies to improve NUE. Among them, the N sources and fertilizer types remarkably affect N losses [7]. The application of enhanced-efficiency fertilizers (EEFs), such as slow- and controlled-release urea (CRU), can control N dynamics through interactions between crops and soils by delaying its release to more closely match it to the nutrient absorption by crops [13]. Zhang et al. (2019) conducted a global meta-analysis and found that a significant reduction in NH_3_ emissions, of 39.4%, and an increase in NUE, of 24.1%, resulted from CRU [14]. The use of urease inhibitors can reduce urease activity, as well as slowing urea hydrolysis, leading to a reduction in NH_3_ volatilization [15]. A decrease of approximately 53% in NH_3_ loss as a result of applying NBPT-treated urea, as opposed to urea, was demonstrated [11]. As animal husbandry develops rapidly, to reduce chemical fertilizer use, increase soil fertility, and alleviate environmental deterioration, organic fertilizers obtained from plant residues and animal waste have been widely used. The possible reduction in N losses by delaying organic N mineralization resulted in significant reductions in the NH_3_ volatilization loss of 8.8–12.7% [8].

Since agricultural biomass in China is produced in large quantities, the country witnesses the production of a tremendous amount of energy crops [16]. As a conservation tillage strategy, straw returning to the field is widely practiced in agricultural ecosystems in China because it can prevent large-scale straw burning in open fields and the resultant air pollution, reduce soil erosion, optimize soil moisture, enhance soil carbon sequestration, improve soil quality, and increase biological activities and crop yields [16,17]. The impact of straw use on NH_3_ volatilization, which is determined by soil properties and straw application rates, however, remains a controversial issue. In previous research, the promotion of NH_3_ volatilization by straw return occurred by increasing the soil NH_4_^+^-N content through straw decomposition [18,19,20]. Straw return at higher C/N ratios, in contrast, may mitigate NH_3_ emissions by promoting the microbial immobilization of the available N and enhancing denitrification [21,22]. The independent effects of straw return and the application of various fertilizer types on NH_3_ volatilization, crop yield, and NUE have been reported. However, to our knowledge, there is limited research on the analysis of the comprehensive effects of straw return and various N fertilizers (such as inhibitors and novel fertilizers) on agronomic and environmental factors.

The purple rock series of the Jurassic and Cretaceous periods, which can be mostly found in the Sichuan Basin, a crucial agricultural region in southwestern China, are the origin of purple soil [23]. Purple soil farmland accounts for 36.5% of the total farmland in the Sichuan Province [17], and the predominant cropping system in hilly regions in the middle of the Sichuan Basin is the oilseed rape (*Brassica napus* L.)–maize (*Zea mays* L.) rotation system. With the rapid development of Chinese society, it is vital to ensure that the labor supply, food security, and environmental protection practices undergo a significant shift. So far, there is a lack of an integrated evaluation of the impacts of straw mulching practices and the application of novel N fertilizers on crop yield and NH_3_ volatilization, especially in purple soil regions in China. However, field-scale experiments should be conducted to confirm whether these management measures contribute to the increase in crop production and the decrease in environmental costs. Therefore, a two-year field trial involving subplots was carried out from 2021 to 2023 in dryland purple soil in southwestern China, a region where oilseed rape–maize rotation is typically practiced. The objectives of the present work are as follows: (1) to assess the responses of NH_3_ volatilization, oilseed rape and maize yields, and NUE to straw mulching and the application of various N fertilizer types and (2) to propose the optimum combination of straw mulching and N fertilizer application as an effective strategy to achieve the sustainable production of oilseed rape and maize in southwestern China.

## 2. Results

### 2.1. Ammonia Volatilization Rates and Cumulative Ammonia Volatilization Losses During the Oilseed Rape and Maize Growing Seasons

**Figure 1:**
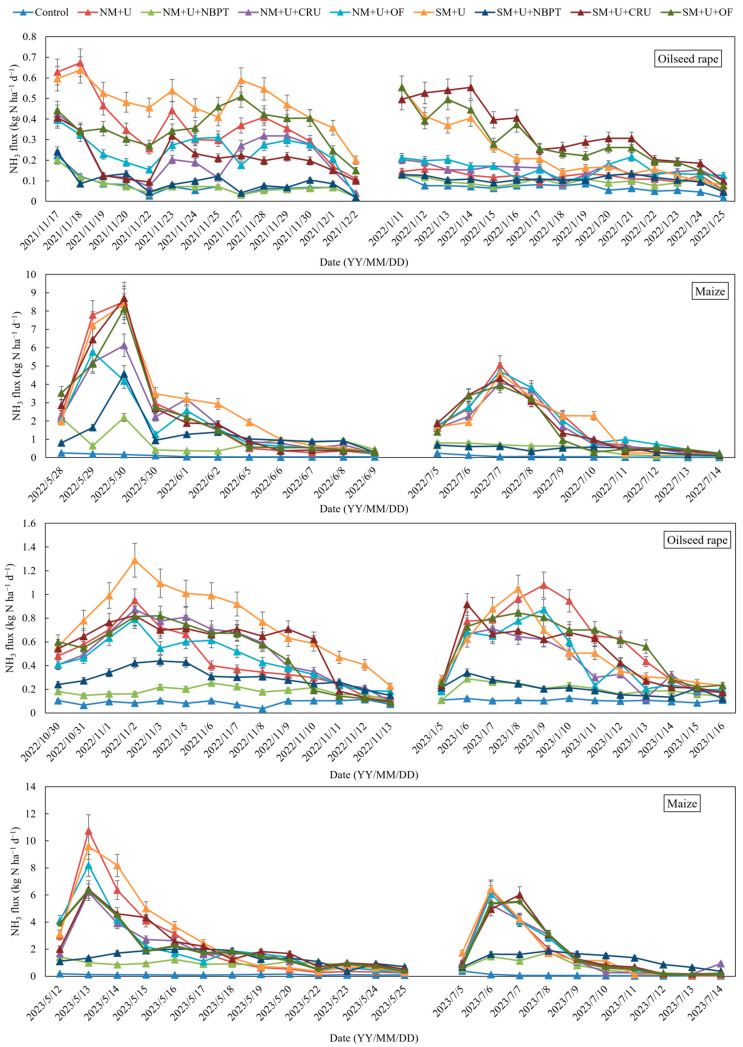
The dynamics of NH_3_ fluxes under different straw mulch and N fertilizer treatments during the oilseed rape- and maize-growing seasons between 2021 and 2023; error bars indicate the standard deviations of the means (*n* = 3).

Due to the application of irrigation after fertilization, the urea granules can be dissolved, followed by hydrolysis which causes the formation of OH^−^ and NH_4_^+^ through ammonification within a short period, leading to an abrupt increase in NH_3_ volatilization. Upon the application of N fertilizers, the trend obtained for NH_3_ volatilization fluxes in all the treatments was quite similar, showing an initial increase and then a decrease. Figure 1 demonstrates that after a first peak at 2–4 days of N application, NH_3_ fluxes rapidly declined to lower levels on the 14th day. Moreover, apparent increases in NH_3_ fluxes were observed during the maize growing season, compared to the oilseed rape growing season. Generally, the maize growing season showed the maximum NH_3_ flux rate of 10.74 kg ha^−1^ d^−1^, whereas, in the oilseed rape growing season, the maximum rate of 0.95 kg ha^−1^ d^−1^ was obtained. Higher peaks were achieved by basal fertilization than by top-dressing fertilization during the two growing seasons. Meanwhile, NH_3_ flux had its maximum value in NM + U, showing significant increases compared with NM + U + NBPT, NM + U + CRU, and NM + U + OF, particularly to a larger extent in the first treatment (*p* < 0.05). Straw-mulching treatments exhibited a similar pattern for NH_3_ fluxes. The absence of N led to low NH_3_ fluxes in both seasons.

NH_3_ volatilization markedly increased following the N fertilization treatment compared with the control treatment. Furthermore, with N fertilization, NH_3_ volatilization losses ranged from 1.35% to 9.29% during the oilseed rape-growing season, while in the maize season, the range of 5.82–21.44% was recorded (Table 1). NH_3_ volatilization mainly occurred during the basal fertilization periods, accounting for 52.34–76.64% and 36.87–74.01% of that for the whole maize-growing and oilseed rape-growing seasons, respectively.

Mean cumulative NH_3_ volatilization losses of 56.34, 20.35, 49.38, and 50.22 kg N ha^−1^ were found under the NM + U, NM + U + NBPT, NM + U + CRU, and NM + U + OF treatments, respectively, over the two years of crop rotation. Compared to the only urea treatment (NM + U), the declines in mean NH_3_ volatilization losses in the NM + U + NBPT, NM + U + CRU, and NM + U + OF treatments were 64.13% (*p* < 0.05), 17.39%, and 15.09% during the oilseed rape season, while the maize season exhibited the values of 64.01% (*p* < 0.05), 11.67%, and 10.13%, respectively.

The SM + U, SM + U + NBPT, SM + U + CRU, and SM + U + OF treatments, however, displayed mean cumulative NH_3_ volatilization losses of 64.53, 29.28, 56.50, and 54.32 kg N ha^−1^, respectively, in two years. Straw mulching combined with the various N fertilizer types caused an increase in NH_3_ volatilization, compared with fertilization application. The NM + U, NM + U + NBPT, NM + U + CRU, and NM + U + OF treatments contributed to increases in the seasonal NH_3_ volatilization losses of 35.65%, 27.45%, 32.83%, and 40.98% during the oilseed rape season, but during the maize season, increases of 10.69%, 45.27%, 12.08%, and 3.00% were noted, respectively.

In comparison with NM + U, NBPT addition resulted in the lowest NH_3_ emission factor, with a decreasing range of 4.39% to 0.77% (*p* < 0.05) in the oilseed rape season and 17.7% to 5.87% in the maize season (*p* < 0.05). The NH_3_ emission factor increased after straw mulch application in the two growing years. The best emission factor for the two years was notably obtained for the straw mulching plus U treatment (SM + U), showing values of 6.19% and 19.64% during the oilseed rape and maize seasons, respectively.

### 2.2. Environmental and Soil Conditions

**Figure 2:**
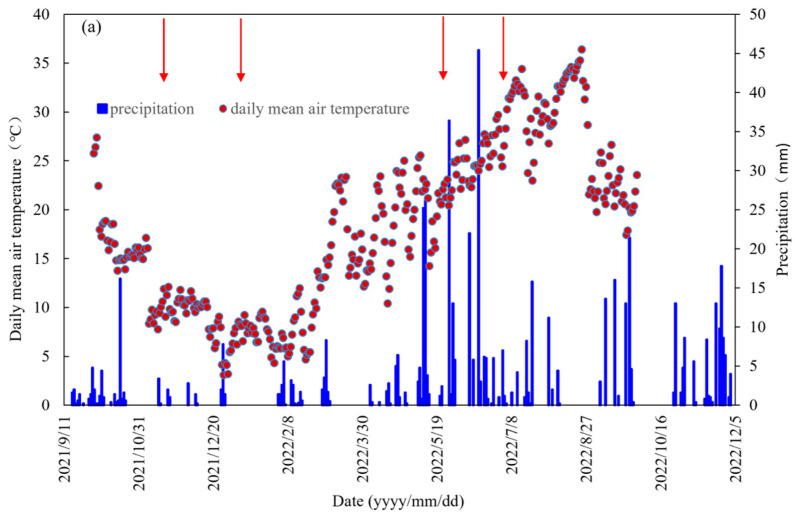

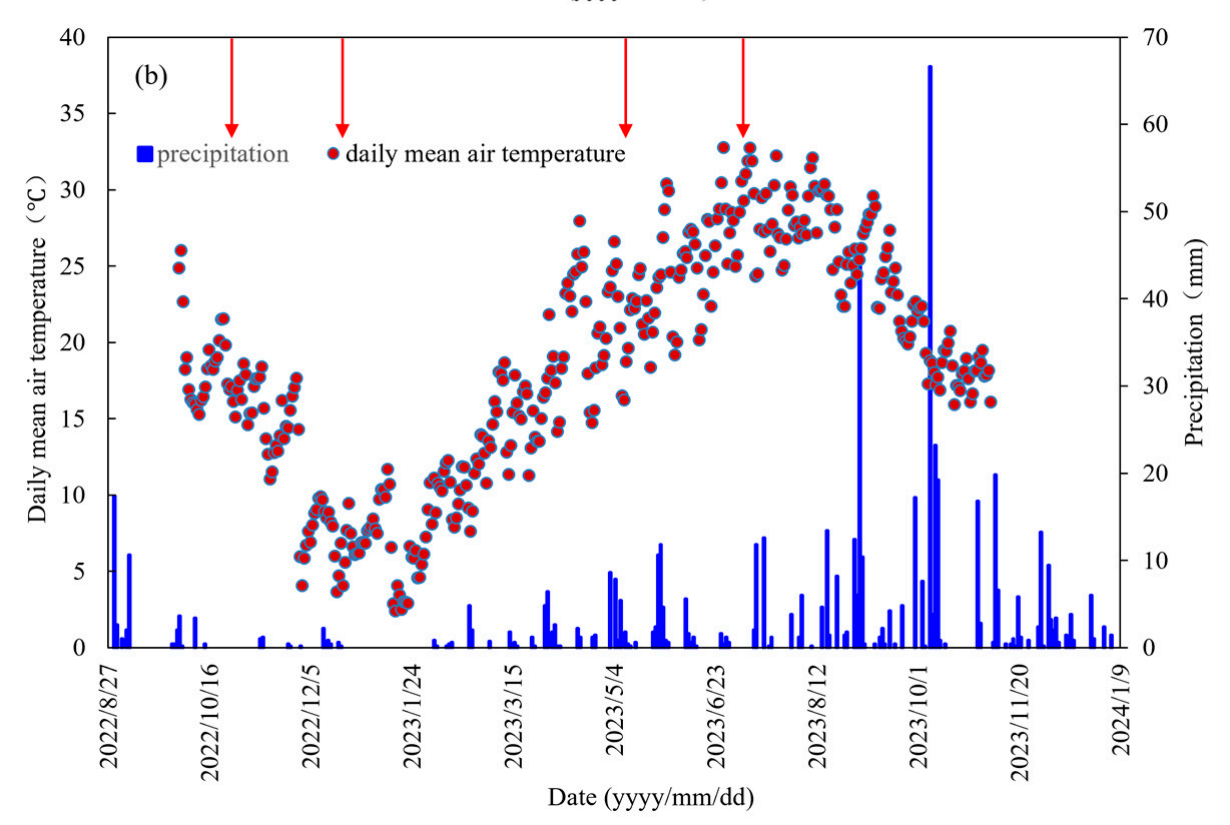
Daily mean air temperature and precipitation in the oilseed rape–maize rotation system at the experimental site from September 2021 to September 2022 (**a**), October 2022 to December 2023 (**b**); red arrows indicate the basal- and top-dressing fertilizers in the oilseed rape and maize growing seasons.

During the basal- and top-dressing fertilization periods, the mean air temperature had values of 10.3 °C and 8.0 °C in the oilseed rape season but 24.3 °C and 32.0 °C in the maize season between 2021 and 2022, whereas from 2022 to 2023, the mean air temperatures were 16.7 °C and 8.2 °C in the oilseed rape season and 22.1 °C and 29.4 °C in the maize season, respectively (Figure 2). The total precipitation values during the evaluation periods amounted to only 8.0 mm and 4.0 mm during the oilseed rape season in 2022 and 2023, respectively. In 2022, the basal fertilizer application preceded a rainfall of 7 mm and six rainfall events, with a total rainfall amount of 16.4 mm in the maize season, while the top-dressing application preceded the first 8 days of the dry period. In 2023, however, the basal fertilization preceded continuous precipitation, with a total amount of 32.6 mm in the last 7 days of the maize season. On the 7th, 8th, and 10th days after N fertilization, precipitation values of 5.6, 1.6, and 1.2 mm were recorded, respectively. For the ammonia flux measurement following top-dressing fertilization, precipitation continued until the measurements were completed, which marked the end of the NH_3_ volatilization process.

**Figure 3:**
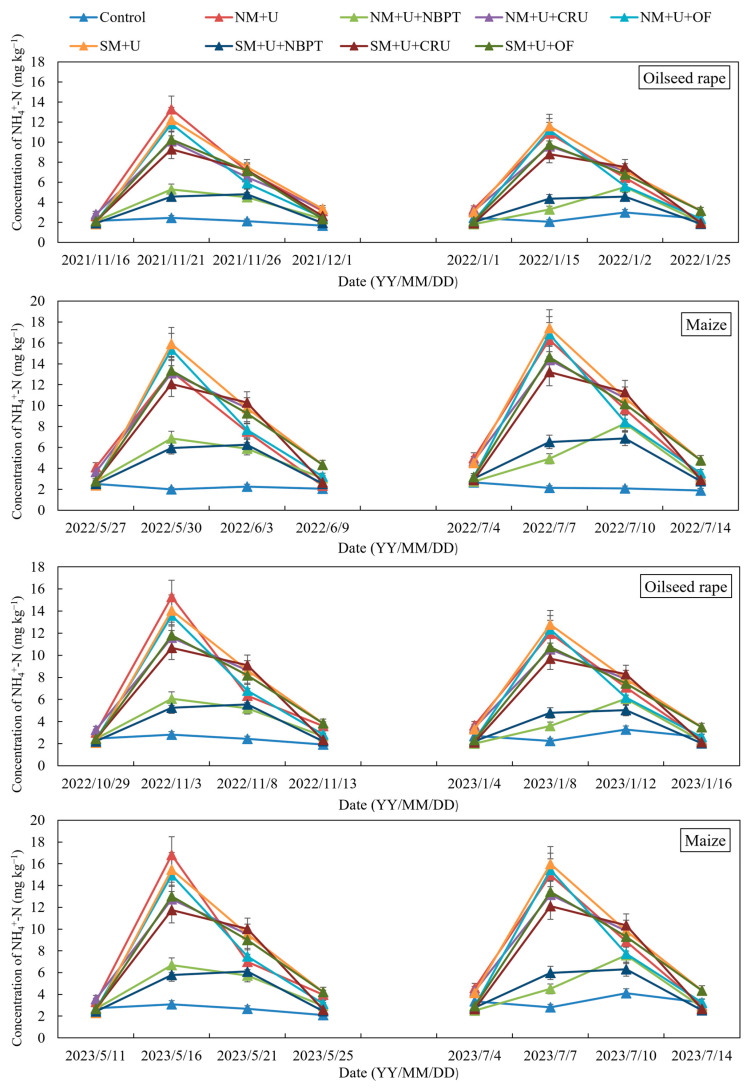
Changes in NH_4_^+^-N concentrations in the topsoil layer (0–20 cm) under different straw mulch and N fertilizer treatments in the oilseed rape and maize growing seasons during the 2021–2023 period; error bars represent standard deviations of the means (*n* = 3).

Because of the irrigation applied after fertilization, urea could be rapidly dissolved and incorporated into the 0–20 cm soil layer. During the warm experimental period, urea was rapidly hydrolyzed, and differing patterns were observed for NH_4_^+^-N concentrations at the 0–20 cm soil depth under different treatments. NH_4_^+^-N was significantly increased by fertilization under the mulching treatments (Figure 3). NH_4_^+^-N concentrations remarkably increased after 5 days of fertilization but decreased thereafter to the levels in soils receiving no N fertilizers on the 10–14th day of each fertilizer application, with the NBPT, CU, and OF treatments showing low peaks and slow declines. Previous evidence demonstrated that the soil NH_4_^+^-N concentration reached its lowest value in U + NBPT, leading to dramatic decreases in NH_3_ volatilization [24]. CRU caused a decrease in the NH_4_^+^ diffusion rate in the soil, upon which NH_3_ volatilization markedly decreased [25]. NH_3_ volatilization under U + OF and U + CRU was almost similar (*p* > 0.05). In the N fertilizer plus straw-mulching treatment, the soil NH_4_^+^-N contents slightly increased compared to those in the treatment of no straw application, with no significant difference between these two treatments.

### 2.3. Grain Production, NUE, and Yield-Scaled NH_3_Volatilization

The oilseed rape production was 1.07–2.99 t ha^−1^, whereas that of maize stood at 2.40–6.35 t ha^−1^ under the different treatments (Table 2). The production of both oilseed rape and maize remarkably increased under the N fertilizer treatments compared to the control, irrespective of straw application. Compared with the sole urea treatment (NM + U), the increases in oilseed rape yield in the NM + U + NBPT, NM + U + CRU, and NM + U + OF treatments were within the ranges of 2.52–5.68% in 2022 and 2.54–5.70% in 2023, while for maize yield, it increased by 1.14–2.64% and 1.08–3.21% in 2022 and 2023, respectively. In comparison with the N fertilization alone, following the combination of straw mulching and various N fertilizers, the increase in oilseed rape yield varied from 3.23% to 4.35% and 3.23% to 4.47% in 2022 and 2023, respectively, whereas that of maize exhibited increases by 0.20–2.28% in 2022 and 1.66–2.08% in 2023. However, all the fertilizer treatments were not significantly different in terms of crop yield. Similarly, there was no obvious difference in oilseed rape or maize production between the straw-mulching treatments.

During the oilseed rape season, when straw mulching was not applied, a non-significant difference in N uptake was obtained between the urea treatment alone and other N fertilizer treatments. During the maize season, however, compared with NM + U, with the addition of NBPT and/or straw mulching, N absorption by maize plants significantly increased (*p* < 0.05).

The NUE for oilseed rape production ranged from 31.26% to 43.35%, whereas for maize, it was within the range of 28.31% to 34.63%. When compared with NM + U, the difference in NUE between the NM + U + NBPT, NM + U + CRU, and NM + U + OF treatments was not significant in the oilseed rape season. NUE was around 4.27% higher in NM + U + NBPT during the maize season (*p* < 0.05). In the SM + U + NBPT, SM + U + CRU, and SM + U + OF treatments, NUE showed 2.61% to 9.29% and 1.25–7.81% higher values during the oilseed and maize seasons, respectively, compared to SM + U. Neither N fertilizer types nor straw mulching influenced nitrogen partial factor productivity (NPFP) in both the oilseed rape and maize seasons. Compared with NM + U, under the NM + U + NBPT, NM + U + CRU, and NM + U + OF treatments, consistently lower values of yield-scaled NH_3_ volatilization were noted, 13.15–65.66% in the oilseed rape season and 10.34–67.27% in the maize season. Straw mulching led to an increase in yield-scaled NH_3_ volatilization over the two growing years. SM + U achieved the maximum yield-scaled NH_3_ volatilization over two years (4.84 and 8.35 kg N t grain^−1^ during the oilseed rape and maize seasons, respectively). Additionally, yield-scaled NH_3_ volatilization underwent a remarkable decrease over these two years (*p* < 0.05) after NBPT addition, compared to SM + U.

## 3. Discussion

### 3.1. Roles of Straw Mulching in Driving NH_3_Volatilization in the Oilseed Rape–Maize System

The implementation of conservation agricultural practices for promoting straw maintenance and its return to the soil surface is increasing in agricultural areas. Straw return has been found to enhance soil fertility while reducing the N fertilization input [26,27]. However, Pan et al. performed a meta-analysis [28] and observed an average increase of 25.5% in NH_3_ volatilization with straw addition compared to bare soil (control). For the two experimental years, following the application of straw mulch combined with various N fertilizers, the annual cumulative NH_3_ volatilization increased by 8.94–55.59% in comparison with fertilization alone, which is similar to the observations made by Zheng et al. [24] and Cheng et al. [29]. Straw residues at a higher C/N ratio applied onto the soil surface can boost urease activity in addition to increasing soil NH_4_^+^-N concentrations, consequently inducing an increase in NH_3_ volatilization [24,30]. Nonetheless, the suppression of soil NH_3_ volatilization to the atmosphere caused by straw can partially compensate for its increase induced by straw mulching [20]. Moreover, under straw mulching, soil temperature increased in winter but decreased in summer, compared with that in conventional flat planting [31]. Consequently, the difference in NH_3_ volatilization between non-mulching and straw mulching with the same N fertilizer type was not significant in some cases. Compared with the oilseed rape season, during the maize season, NH_3_ volatilization loss increased to a greater extent under straw mulching. The NH_4_^+^-N content and urea hydrolysis in the soil may increase as a result of incomplete straw decomposition from the last oilseed rape season due to the increase in microbial biomass for several months and higher soil temperatures [24].

Zheng et al. [24] reported the improving effect of straw mulching on maize production by 6.2% and 4.7% in 2018 and 2019, respectively, when compared with non-mulching. Hu et al. [31] found that straw mulching increased maize yield by 59.6% in 2014 and by 28.9% in 2015. In the present study, compared with the no-mulching treatment, straw-mulching treatments resulted in an increase in crop yield of 3.23–4.47% and 0.20–2.28% for the oilseed rape and maize seasons, respectively. Crop production and NUE were not significantly different across the four N types, despite the straw mulching. This result may be due to the lower seasonal precipitation in our work, 365 mm and 402 mm during the 2021–2022 and 2022–2023 periods, respectively, compared to the values reported by a previous study (417 mm in 2018 and 500 mm in 2019) [24], which indicates the adverse impact of soil mulching on straw degradation to increase soil organic carbon. Zhang et al. [32] concluded that straw mulching apparently increased crop production compared to conventional fertilization until the 3rd year of the experiment.

### 3.2. Roles of Different N Fertilizer Types in Driving NH_3_Volatilization in the Oilseed Rape–Maize System

In farmland ecosystems, urea as a representative N fertilizer can be the major source of ammonium ions and is converted to NH_3_. Since the application of fertilizers is usually followed by mild irrigation in the study region, the higher relative humidity at ground level promoted the dissolution of urea granules, which is attributable to urea hydrolysis catalyzed by urease [20]. Cumulative NH_3_ volatilization amounted to 2.43–16.72 and 14.55–53.60 kg N ha^−1^, accounting for 1.35–9.29% and 5.82–21.44% of the total N fertilizer applied during the oilseed rape and maize seasons, respectively. The seasonal NH_3_ volatilization values obtained in our study were close to those previously found in the dryland in China [12,32,33].

The results of an ANCOVA analysis showed a significant correlation between the NH_3_ volatilization, growing year, straw treatments, and N fertilizer types (*p* < 0.05; Table 3). The significant impact of NBPT on NH_3_ volatilization loss, with an average decrease of 64.0% upon urea application in the present work, is consistent with the result of other research reporting decreases ranging from 47.0% to 89.0% in different soil types, cropping systems, and climate regions, as well as under different N addition rates [34,35]. A reduction in NH_3_ emissions of11.67–17.19% was caused by the NM + U + CRU treatment, compared to NM + U. This result conforms to the findings presented by Zheng et al. [24], who revealed that the use of CRU decreasedNH_3_ emissions by 14.6–42.3% in a dryland maize field in northwestern China. The application of organic fertilizers instead of their chemical counterparts also resulted in a decrease in NH_3_ volatilization losses. Compared with NM + U, NH_3_ emissions were reduced by 10.13–15.09% under the NM + U + OF treatment. The decomposition of organic fertilizers may induce the production of organic acid, decrease the soil pH, improve the NH_4_^+^ adsorption, and increase the soil water holding capacity, ultimately reducing NH_3_ emissions [12]. Generally, the effective minimization of NH_3_ volatilization losses by NM + U + NBPT was manifested by the markedly lowest mean emission factor of NH_3_ observed over two years (3.74%); the mean emission factor under NM + U + CRU was 10.49% and under NM + U + OF was 10.68%, whereas the NM + U treatment exhibited the highest emission factor (12.11%). The order of NM + U > NM + U + OF≈NM + U + CRU > NM + U + NBPT was shown for total NH_3_ volatilization losses under various N-fertilizer treatments.

The increase in crop grain yield under the NM + U + NBPT, NM + U + CRU, and NM + U + OF treatments ranged from 1.08% to 5.69%, but with no significant difference among them (*p* > 0.05), compared to NM + U. A marked increase in N absorption of 9.19% (*p* < 0.05) for the U + NBPT treatment during the maize season, but not during the oilseed rape season, was demonstrated. Likewise, Singh et al. [36] found an apparent decrease in NH_3_ volatilization by urease inhibitor, while the herbage yield only mildly increased. Yang et al. [37], similarly, reported that urease inhibitor decreased NH_3_ volatilization but caused no significant change in crop production. The non-limiting background soil N level, which probably masks the effects of N exerted by inhibitors and novel fertilizers on crop development, may partly explain the above-mentioned result [38]. Besides, crop growth responses to these inhibitors and novel fertilizers could not be identified, probably because of other limiting edaphic variables, such as soil moisture and additional nutrients, or environmental factors, including temperature and precipitation [39,40].

### 3.3. Interannual Variations of NH_3_Volatilization Fluxes

The level and type of fertilizers applied onto the soil surface affected NH_3_-N losses, irrespective of the experimental year, and after fertilization, the dynamics of NH_3_ volatilization rates were greatly affected by climatic factors, particularly the intensity and distribution of precipitation and temperature. NH_3_ may be volatilized from the soil under rainy and low-temperature conditions [17,41].

During the oilseed rape growing period, seasonal precipitation was generally low, and rare heavy rainfall occurred after crop cultivation and fertilization in the study region. In this study, less NH_3_ volatilization being observed during the first oilseed rape cropping season when compared to that observed during the second season is mainly due to the lower atmospheric temperature following the fertilizer application. N fertilizers were mostly utilized as a basal dressing, with high mean air temperatures of 10.3 °C in 2021 and 16.7 °C in 2022 in the period in comparison to the top-dressing fertilizer period, which exhibited values of 8.0 °C and 8.2 °C in 2021 and 2022, respectively. During the entire oilseed rape season, a decrease in NH_3_-N losses was recorded in the top-dressing fertilizer period, which is probably attributable to a higher crop N demand at the bolting stage and a lower mean air temperature. The suppression of urea hydrolysis and microbial activity, possibly by cold weather [30], induced an evident reduction in ammonia volatilization peaks during the first season compared with those during the second one.

Our results revealed that in the first maize growing season, between 10 May 2022 and 25 August 2022, the mean precipitation was 206.4 mm, with a total amount of 97.8 mm after cultivation and fertilization. During the second maize growing season, from 4 May 2023 to 20 August 2023, precipitation had a mean value of 226.8 mm, but it was only 61.4 mm following both cultivation and fertilization. Furthermore, from 1 May to 14 July in 2023, an increase in mean temperature of 0.5 °C was witnessed compared with that recorded in 2022. Cumulative NH_3_ volatilization under the NM + U, NM + U + NBPT, NM + U + CRU, and NM + U + OF treatments in the maize season in 2023 showed increases of 10.1%, 29.1%, 6.5%, and 16.7%, respectively, when compared to the case in 2022. The higher air temperature and lower precipitation after fertilization may pose a higher risk of NH_3_ loss [7,42].

### 3.4. The Optimal Combination of Straw Mulching and Fertilization Under the Oilseed Rape–Maize Rotation System

Straw return has been regarded as an effective approach to utilizing straw resources. The challenge of promoting NH_3_ volatilization by using straw return was solved by implementing optimal N fertilizer management practices [16]. The positive responses of NH_3_ volatilization to straw mulching decreased under NBPT, CRU, and OF in the present study. The promoting effects of straw mulching on the annual NH_3_ volatilization under the U treatment were estimated at 16.79% and 15.73% in 2021–2022 and 2022–2023, which were higher than those under the NBPT, CRU or OF treatments by 62.36%, 12.87%, and 14.94% in 2021–2022, and 48.17%, 12.07%, and 16.55% in 2022–2023, respectively. These results are in agreement with those published by Zheng et al. [22] and Wang et al. [14], who reported that NH_3_ volatilization was markedly reduced after straw mulching integrated with NBPT or CRU compared with that following the application of urea alone, which is probably due to the reduced soil NH_4_^+^-N concentrations.

In the present study, the average NH_3_ volatilization from the summer maize field in the NM + U treatment group over the two growing years was 46.15 kg N ha^−1^, higher than the value obtained by Zheng et al. [24] and Zhang et al. [43]. Consequently, the N fertilization of the purple soil for increasing dryland crop yield may minimize NH_3_ losses, and promoting the advances in fertilizer technologies can be effective in reducing NH_3_ volatilization. First, straw mulching, along with NBPT mixed with urea, was found to be the strategy that could effectively reduce NH_3_ volatilization losses. Second, the application of slow-release N fertilizers or organic fertilizers, which can partially replace chemical fertilizers, likely causes a reduction in N losses. Moreover, urea should be sidedressed before rain. It is noteworthy that the combination of fertilizers with controlled-release coatings and urease inhibitors has not been extensively practiced, despite their role in the effective mitigation of NH_3_ volatilization from different agricultural systems. However, the main obstacle to the popularity of such fertilizer products is that their additional costs cannot guarantee the benefits to plant productivity [44]. Funding these products to offset the additional costs inflicted upon farmers, particularly in areas where there is an increased risk of NH_3_ exposure to humans and ecosystems, can increase their use.

## 4. Materials and Methods

### 4.1. Site Description

**Figure 4:**
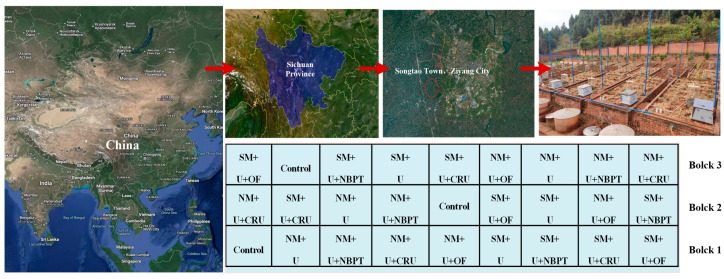
Study area and field plot layout showing the experimental design.

The experimental site in our study is located in Songtao Town, Yanjiang District, Ziyang City (104°34′12″~104°35′19″ E, 30°05′12″~30°06′44″ N), Sichuan Province, China (Figure 4). It is dominated by a moderate humid subtropical monsoon climate and has an annual mean temperature and precipitation of 16.8 °C and 831.86 mm, respectively, with 80% of the latter occurring from June to September. Purple soils are widely distributed in this area. According to US Taxonomy, purple soil is the most common soil type categorized into Entisol. It is typically found at a depth of 50–80 cm, with low soil productivity and light texture [17].

The experiment was conducted on a runoff subplot with a length and width of 4 m and 2 m, respectively, and a slope of 8°. Sampling in this subplot was carried out from the soil profile layer of 0–60 cm. The initial characteristics of the topsoil (0–20 cm) before starting the experiment were as follows: an organic matter (OM) content of 9.05 g kg^−1^, a total nitrogen (TN) content of 0.73 g kg^−1^, an available potassium (AK) content of 171 mg kg^−1^, an available phosphorus (AP) content of 19.8 mg kg^−1^, a pH of 7.47, and a bulk density of 1.24 g cm^−3^. Figure 2 presents the daily mean air temperature and precipitation values under the oilseed rape–maize rotation system.

### 4.2. Experimental Design

This experiment was laid out in a randomized complete block design (RCBD) with 2 factors and 3 replicates. It consisted of two mulching practices, including straw mulching (SM, flat cultivation using the 15 cm long straw harvested from the previous season by evenly spreading it over the soil surface) and non-mulching (NM, flat cultivation without mulching), and 4 N fertilizer types, i.e., urea (U, 46% N); urea (U) combined with a urease inhibitor, N-(n-butyl) thiophosphoric triamide, (U + NBPT);a mixture of urea (U) and controlled-release urea (CRU, 37% N) at a N ratio of 3:7 (U + CRU); and blending U and commercial organic fertilizer at a 3:7 N ratio (U + OF). The NBPT (Agrotain Ultra, Koch Fertilizer LLC, Wichita, KS, USA) was added to the urea at 1% *w*/*w* to form the NBPT-treated urea. The controlled-release urea was provided by Hanfeng Slow-release Fertilizer Co., Ltd., Taizhou, Jiangsu, China. The organic fertilizer was produced by fermenting chicken manure, and its N, *p*, and K total contents were measured before application (2.60%, 3.11%, and 4.38%, respectively).

A total of nine treatments, established for both the oilseed rape and maize seasons, included the following: T0: control (0N); T1: NM + U; T2: NM + U + NBPT; T3: NM + U + CRU; T4: NM + U + OF; T5: SM + U; T6: SM + U + NBPT; T7: SM + U + CRU; and T8: SM + U + OF. They were all replicated thrice. Based on the local farming practices, where irrigation is expected after fertilization, irrigation was not applied during either of the growing seasons in our study. Weed, disease, and insect control were carried out by adopting plant protection strategies, like artificial weeding.

Both the full amounts of the maize and oilseed rape straw were returned to the field. N fertilizer was applied at 180 and 250 kg N ha^−1^ for oilseed rape and maize, according to the Fertilizer Recommendations for Major Crops in China [45], respectively, which were split between basal- and top-dressing applications at a ratio of 6:4. The basal fertilizers were calcium superphosphate and potassium oxide, applied at 90 kg P_2_O_5_ ha^−1^ and 75 kg K_2_O ha^−1^ for oilseed rape and 125 kg P_2_O_5_ ha^−1^ and 75 kg K_2_O ha^−1^ for maize, respectively. The application of the top-dressing fertilizer was completed at the bolting and stem elongation stages of the oilseed rape and maize, respectively.

The oilseed rape cultivar used in this study was “Chuanyou 83”, which has been widely grown in southwestern China. During the first rotation year, oilseed rape was sowed on 20 September, transplanted on 25 October 2021, and harvested on 5 May 2022, while during the second year, its transplanting was carried out on 10 October 2022, and it was harvested on 1 May 2023. Both the plant spacing and row spacing were 50 cm, with a planting density of 40,000 plants ha^−1^. “Zhenghong 6” was the used maize cultivar, which was transplanted and harvested on 10 May and 25 August in 2022, respectively, while in 2023, its transplantation was carried out on 4 May and harvesting was carried outon 20 August. The plant and row spacings and the planting density were 25 and 80 cm and 50,000 plants ha^−1^, respectively. Spray irrigation was performed immediately after fertilization using the center-pivot irrigation system at 5 mm per event.

### 4.3. The Determination of NH_3_Volatilization

NH_3_ volatilization was determined according to the continuous air-flow enclosure method [30,43] using a transparent Plexiglass chamber with an inner diameter of 15 cm and height of 20 cm, which was placed 10 cm deep into the soil. The determination of NH_3_ fluxes was carried out twice daily between 8:00 and 10:00 and between 15:00 and 17:00. Air was pumped via an outlet through a chamber acid-trap system containing 60 mL of the 20% (*w*/*w*) boric acid solution as an adsorbent for the capture of the atmospheric NH_3_, and then, using a pump, the air exchange rate in the test chamber was set at 20% of the chamber volume per min. The concentration of NH_3_ captured in the acid trap was measured by titration with 0.01M H_2_SO_4_, with a bromocresol green-methyl red solution in ethanol as the indicator. Immediately after the capture, the NH_3_ concentration in the absorbent solutions was determined. NH_3_ fluxes were measured daily, after each fertilizer application, between 17 November and 2 December 2021; between 11 January and 25 January, 28 May and 9 June, 5 July and 14 July, and 30 October and 13 November in 2022; and between 5 January and 16 January, 12 May and 25 May, and 5 July and 14 July in 2023. The cumulative NH_3_ loss (kg ha^−1^) in every treatment was calculated by adding up all the obtained daily volatilization rates.

### 4.4. Soil and Plant Sampling

Soil surface (0–20 cm) samples collected from five different positions in each plot were thoroughly mixed to form a composite sample from which to measure the NH_4_^+^-N content. After the freezing and thawing of the collected soil samples, NH_4_^+^-N was extracted using a1 M KCl solution, and its content was analyzed using the TRACCS 2000 (Seal, Homburg, Germany) continuous flow analyzer.

All the oilseed rape and maize plants from each subplot were collected at the harvesting stage for the yield measurement. Five oilseed rape and maize plants, sampled separately from each subplot, were used to determine the differences in N uptake between the straw mulching and various N fertilizer treatments. The plant tissues were digested with a mixture of concentrated sulfuric acid and hydrogen peroxide to determine the total N in plant materials using a continuous flow analyzer (TRACCS-2000). Thereafter, the NH_3_ emission factor, NUE, yield-scaled NH_3_ emissions, and nitrogen partial factor productivity (NPFP) were determined according to the method described by He et al. [46] and Dong et al. [38].

The NH_3_ emission factor of the applied N (%) was calculated as follows:

Emission factor (%) = (E_fertilizer_ − E_control_)/N applied (1)

where E_fertilizer_ and E_control_ denote the cumulative NH_3_ emissions (kg N ha^−1^) from the N fertilizer and control treatments, respectively.

The yield-scaled NH_3_ emissions (kg N t^−1^ grain) were calculated as follows:

Yield-scale NH_3_ emission = cumulative NH_3_ emission/yield (2)

The NUE was calculated by dividing differences in the N uptake amount in the aboveground biomass between N-fertilized and control treatments by the N application rate. The NPFP was the ratio of grain yield and N applied.

### 4.5. Statistical Data Analysis

The data collected during the oilseed rape and maize seasons were analyzed. OriginLab 2017 was used to create figures. To test the significance of the differences among the various treatments for the crop yield, NH_3_ volatilization, NH_3_ emission factor, NUE, nitrogen partial factor productivity (NPFP), and yield-scaled NH_3_ emissions, the analysis of variance (ANOVA) and the least significant difference (LSD) test at the 5% level of significance were used. SPSS Statistics 26 (SPSS, IBM, Armonk, NY, USA) was employed to perform the statistical analysis.

## 5. Conclusions

The present work provides a comprehensive evaluation of the effects of the combination of straw mulching and four different N fertilizer types on the NH_3_ volatilization, grain yield, and NUE of oilseed rape and maize. The results revealed remarkably increased cumulative NH_3_ volatilization as a result of straw mulching during the oilseed rape and maize seasons, but crop production and NUE were not markedly affected. The NBPT application mitigated NH_3_ volatilization to the greatest extent across the different N fertilizer types, probably because of the decrease in the soil NH_4_^+^-N content. Crop production and total N adsorption were promoted by the co-application of straw mulching and NBPT, which led to a significant reduction in yield-scaled NH_3_ volatilization. Therefore, straw mulching combined with the mixture of NBPT and urea was found to be the optimal agricultural practice to improve crop production and NUE and reduce N losses in sustainable agriculture. Future research will focus on the impacts of straw mulching and the application of novel N fertilizers on soil labile N and C pools, the relationship between soil nutrients and NH_3_ volatilization, as well as the integrated observation of N loss, including NH_3_ volatilization, denitrification, leaching and runoff.

## Figures and Tables

**Table 1:** Cumulative ammonia volatilization, N-fertilizer losses, and ammonia emission factors after basal- and top-dressing fertilization from 2021 to 2023.

Year	Crop Season	Treatment	Basal Dressing		Top Dressing		Cumulative Losses (kg ha^−1^)	Total Loss Rate (%)	NH_3_ Emission Factor (%)
			Nitrogen Loss (kg ha^−1^)	Loss Rate (%)	Nitrogen Loss (kg ha^−1^)	Loss Rate (%)			
2021–2022	Oilseed rape	T0	1.05 d	-	1.02 e	-	2.07 e	-	-
		T1	5.11 b	4.73 b	1.79 cde	2.49 cd	6.90 c	3.83 c	2.68 c
		T2	1.03 d	0.95 d	1.40 de	1.94 d	2.43 e	1.35 e	0.20 e
		T3	2.96 c	2.74 c	2.25 cd	3.12 cd	5.21 d	2.89 d	1.74 d
		T4	3.43 c	3.18 c	2.36 c	3.28 c	5.79 d	3.22 d	2.07 d
		T5	6.67 a	6.17 a	3.49 b	4.84 b	10.16 a	5.64 a	4.49 a
		T6	1.32 d	1.23 d	1.60 cde	2.22 cd	2.92 e	1.62 e	0.47 e
		T7	2.93 c	2.71 c	5.01 a	6.96 a	7.93 b	4.41 b	3.26 b
		T8	5.00 b	4.63 b	4.38 a	6.08 a	9.38 a	5.21 a	4.06 a
2021–2022	Maize	T0	1.05 d	-	0.68 d	-	1.74 c	-	-
		T1	26.98 ab	17.99 ab	16.96 ab	16.96 ab	43.94 a	17.58 a	16.88 a
		T2	10.10 cd	6.73 c	4.46 c	4.46 c	14.55 bc	5.82 b	5.13 b
		T3	23.84 abc	15.89 abc	15.62 ab	15.62 ab	39.46 a	15.78 a	15.09 a
		T4	20.06 abc	13.37 abc	18.27 a	18.27 a	38.33 a	15.33 a	14.64 a
		T5	31.64 a	21.09 a	16.94 ab	16.94 ab	48.58 a	19.43 a	18.74 a
		T6	14.70 bcd	9.80 bc	4.48 c	4.48 c	19.19 b	7.67 b	6.98 b
		T7	26.77 ab	17.85 ab	16.47 ab	16.47 ab	43.24 a	17.30 a	16.60 a
		T8	25.68 ab	17.12 abc	14.90 b	14.90 b	40.58 a	16.23 a	15.53 a
2022–2023	Oilseed rape	T0	1.25 d	-	1.27 b	-	2.51 d	-	-
		T1	6.35 bc	5.88 bc	7.12 a	9.89 a	13.47 ab	7.48 ab	6.09 ab
		T2	2.55 d	2.36 d	2.38 b	3.31 b	4.93 cd	2.74 c	1.34 c
		T3	7.30 bc	6.76 bc	4.78 a	6.64 ab	12.09 b	6.72 b	5.32 b
		T4	6.32 b	5.85 bc	5.24 a	7.28 a	11.56 b	6.42 b	5.03 b
		T5	10.73 a	9.93 a	5.99 a	8.32 a	16.72 a	9.29 a	7.89 a
		T6	4.19 cd	3.88 cd	2.44 b	3.38 b	6.62 c	3.68 c	2.29 c
		T7	7.95 ab	7.37 ab	5.74 a	7.97 a	13.69 ab	7.61 ab	6.21 ab
		T8	7.12 bc	6.59 bc	6.76 a	9.38 a	13.87 ab	7.71 ab	6.31 ab
2022–2023	Maize	T0	1.43 f	-	0.83 f	-	2.26 f	-	-
		T1	32.39 ab	21.59 ab	15.97 b	15.97 b	48.36 b	19.35 a	18.44 a
		T2	11.73 e	7.82 e	7.06 d	7.06 d	18.79 e	7.52 d	6.61 d
		T3	25.71 c	17.14 c	16.30 b	16.30 b	42.01 c	16.80 b	15.90 b
		T4	28.13 bc	18.75 bc	16.62 b	16.62 b	44.75 bc	17.90 ab	17.00 ab
		T5	36.65 a	24.43 a	16.95 ab	16.95 ab	53.60 a	21.44 a	20.54 a
		T6	17.60 d	11.73 d	12.22 c	12.22 c	29.82 d	11.93 c	11.02 c
		T7	29.93 bc	19.95 bc	18.20 a	18.20 a	48.13 b	19.25 a	18.35 a
		T8	27.38 c	18.25 bc	17.42 ab	17.42 ab	44.81 bc	17.92 ab	17.02 ab

Note: Different letters indicate significant differences between the means of the different treatments in the same growing year (*p* < 0.05).

**Table 2:** Crop yields, N uptake, NUE, nitrogen partial factor productivity (NPFP), and yield-scaled NH_3_ emissions in two cycles of oilseed rape–maize rotation between 2021 and 2023.

Cycle	Crop	Treatment	Yield (t ha^−1^)	N Uptake (kg N ha^−1^)	NUE (%)	NPFP (kg kg^−1^)	Yield-Scaled NH_3_ Emission (kg N t^−1^ Grain)
2021–2022	Oilseed rape	T0	1.07 b	69.82 d	-	-	1.93 b
		NM + U	2.61 a	133.43 abc	35.34 abc	14.50 a	2.64 ab
		NM + U + NBPT	2.68 a	134.37 abc	35.86 abc	14.87 a	0.91 c
		NM + U + CRU	2.69 a	131.24 bc	34.12 bc	14.97 a	1.93 b
		NM + U + OF	2.76 a	130.00 c	33.42 c	15.32 a	2.10 b
		SM + U	2.69 a	129.57 c	33.19 c	14.97 a	3.77 a
		SM + U + NBPT	2.79 a	146.29 a	42.48 a	15.51 a	1.05 c
		SM + U + CRU	2.81 a	137.48 abc	37.59 abc	15.60 a	2.83 ab
		SM + U + OF	2.85 a	145.15 ab	41.85 ab	15.83 a	3.29 a
	Maize	T0	2.40 b	41.13 d	-	-	0.72 d
		NM + U	5.92 a	114.65 c	29.41 bc	23.67 a	7.43 a
		NM + U + NBPT	5.99 a	124.17 ab	33.22 a	23.94 a	2.43 c
		NM + U + CRU	6.05 a	114.33 c	29.28 bc	24.21 a	6.52 ab
		NM + U + OF	6.07 a	117.50 bc	30.55 b	24.29 a	6.31 ab
		SM + U	6.05 a	111.90 c	28.31 c	24.21 a	8.03 a
		SM + U + NBPT	6.07 a	127.17 a	34.42 a	24.30 a	3.16 c
		SM + U + CRU	6.06 a	115.03 c	29.56 bc	24.26 a	7.13 a
		SM + U + OF	6.17 a	127.70 a	34.63 a	24.70 a	6.57 ab
2022–2023	Oilseed rape	T0	1.13 b	73.16 d	-	-	2.23 c
		NM + U	2.74 a	137.48 abc	35.73 bcd	15.22 a	4.92 a
		NM + U + NBPT	2.81 a	134.58 bc	34.12 cd	15.61 a	1.75 c
		NM + U + CRU	2.83 a	129.42 c	31.26 d	15.72 a	4.27 ab
		NM + U + OF	2.90 a	136.53 abc	35.21 cd	16.09 a	3.99 b
		SM + U	2.83 a	139.88 abc	37.07 abcd	15.72 a	5.91 a
		SM + U + NBPT	2.94 a	150.43 ab	42.93 ab	16.31 a	2.26 bc
		SM + U + CRU	2.95 a	144.58 abc	39.68 abc	16.38 a	4.64 a
		SM + U + OF	2.99 a	151.19 a	43.35 a	16.62 a	4.64 a
	Maize	T0	2.53 b	43.76 e	-	-	0.89 d
		NM + U	6.05 a	117.51 c	29.50 bc	24.21 a	7.99 a
		NM + U + NBPT	6.16 a	129.35 a	34.23 a	24.65 a	3.05 c
		NM + U + CRU	6.12 a	127.73 a	33.59 a	24.47 a	6.87 b
		NM + U + OF	6.25 a	116.29 cd	29.01 c	24.99 a	7.16 ab
		SM + U	6.18 a	110.30 d	26.61 d	24.71 a	8.68 a
		SM + U + NBPT	6.29 a	122.23 bc	31.39 b	25.15 a	4.74 c
		SM + U + CRU	6.22 a	121.94 bc	31.27 b	24.88 a	7.74 a
		SM + U + OF	6.35 a	129.82 a	34.42 a	25.40 a	7.06 ab

Note: Different letters above every value denote significant differences among the means of various treatments (*p* < 0.05).

**Table 3:** Effects of the growing year (Y), straw mulching (S) treatments, nitrogen fertilizer (N) types, and their interactions on cumulative NH_3_ volatilization.

Factors	Df	SS	F	*p*
Y	1	1221.1	31.87	<0.001
N	4	18,823.0	122.80	<0.001
S	1	602.6	15.72	<0.001
Y × N	4	125.5	0.8185	0.5219
Y × S	1	6.6	0.1727	0.6802
N × S	3	40.5	0.3524	0.7877
Y × N × S	3	47.1	0.4095	0.7471
Residuals	36	1379.5	-	-

Df: degree of freedom; SS: square sum of errors.

## Data Availability

The data are accessible within the article.
